# The Somme: January 1919

**Published:** 1919-02-22

**Authors:** A. Lombardini


### THE SOMME: JANUARY 1919.

By A. LOMBARDINI, C.F.

DESCRIPTIONS and photographs of the battle area convey only a slight impression of the terrible devastation which war has wreaked on a country once so fair and still.

I have just returned from a tour of the Somme battlefields. Nothing can ever remove the picture of utter hopelessness which the scene imprinted on my mind. Towns bordering the fighting zone such as Abbeville and Amiens are scarred, but those within the zone are blasted beyond recognition. The villages of Villers Brettonieux, Abancourt, and Lamotte are sufficiently near to Amiens to allow groups of villagers to visit the places of their birth and childhood. In the majority of cases they do not appear to be able to recognise the house they once inhabited. The streets are one continuous heap of broken brick without mark or character. The tragedy of war could not be more poignantly revealed than in the picture of a little family group dressed in black sitting on a pile of débris sobbing bitterly like broken-hearted children.

Climbing over rafters and masonry in the little church of Wiencourt, I saw under the beams what appeared to be a human hand stained with blood, but a clearance of the dust and stone revealed, not a human body, but a life-size figure of the crucified Lord, wrenched from its Cross by the bursting of the high explosive.

Strong points, sunken roads, and registered targets need no other indication than the groups of crosses and mounds, for hundreds of men lie buried where they fought and fell.

The city of Cambrai is, to all appearances, a German town. During the four years' occupation, apparently no street direction or order was permitted except in the German language. The attempt to burn the town was foiled by the arrival of the victorious armies, but sufficient damage was done in the centre to reveal the intention of the ghouls. The main arteries from town to town are fringed with little wooden crosses, singly and in groups, adorned with belts of machine-gun cartridges, steel helmets, and bayonets. Sometimes only a bottle containing an identity disc marks the resting-place of a hero, but this matter is speedily being dealt with by the Graves Registration Committee, whose work is worthy of the highest praise.

The whole scene is a maze of barbed-wire, trench and dugout, and overhanging the thousand miles of blasted soil is, like a funeral pall, that peculiar odour suggestive of death.

In Peronne, Bapaume, and Albert, towns once so flourishing and happy, there reigns a ghastly silence, for nothing lives except rats, which are indeed plentiful. No sign of a dog, or sound of a bird. Along the roads, the only signs of a château or "residence in its own grounds" are the twisted iron palings and a mound of rubble. Of trees there are none. Splintered stumps mark the lines of pre-war stately avenues, while on either side of what was once a road the hollows of shell craters suggest small-pox in its most virulent form.

In trenches and dugouts there are still the bullet-pierced helmets, cartridge-belts, and shells lying just as they were left, and the mine-craters and blown-up bridges reveal the methodical way in which the Hun tried to stay that historical advance which, if the armistice had not intervened, would have ended in the complete annihilation of his armies. At various intervals during day and night the sound of explosions on the battlefield serve as gruesome reminders that unexploded mines and shells still remain to deal out death to the unwary and venturesome.

This valley of the Somme will remain an imperishable memorial of the valour and endurance of gallant men. To see is to revere and rejoice. All who can should visit this undulating altar of sacrifice. Memorials and flowers, trees and birds, will some day appear, and the gratitude of a redeemed mankind will be directed towards, and form an invisible halo above the sod where heroes rest. But—the Somme will always be the Somme!

